# Innovations in Medical Education During the COVID-19 Era and Beyond: Medical Students' Perspectives on the Transformation of Real Public Health Visits Into Virtual Format

**DOI:** 10.3389/fpubh.2022.883003

**Published:** 2022-06-13

**Authors:** Salman Alzayani, Adel Alsayyad, Khaldoon Al-Roomi, Amer Almarabheh

**Affiliations:** ^1^Department of Family and Community Medicine, College of Medicine and Medical Sciences, Arabian Gulf University, Manama, Bahrain; ^2^Public Health Directorate, Ministry of Health, Manama, Bahrain

**Keywords:** COVID-19, education, field visits, public health, virtual

## Abstract

**Background:**

At the College of Medicine and Medical Sciences (CMMS), Arabian Gulf University (AGU), Bahrain, the Public Health Program comprises two core components, namely, lectures and field visits (consumer products safety, communicable diseases control, and food safety). Digital transformation has innovated the medical educational activities during the COVID-19 pandemic where the real public health field visits were transformed into a virtual format. This study is aimed to examine the potential effect of converting the real public health field visit programs into a virtual format during the COVID-19 pandemic.

**Methods:**

All medical students who have submitted online feedback evaluation forms upon completing the Public Health Program in the academic years 2019–2020 (180 students; before the COVID-19 pandemic) and 2020–2021 (167 students; during the COVID-19 pandemic) were included in the study, a total sample size of 347 responses. Independent samples *t*-test was employed to compare students' feedback on Public Health Program before and during the COVID-19 pandemic while the Pearson chi-square test was used for categorical data. A *p*-value of <0.05 was considered statistically significant.

**Results:**

The mean score of students' satisfaction from the virtual program during the COVID-19 pandemic toward the consumer products safety and food safety field visits was significantly higher than that for students before the COVID-19 pandemic (the real field visits). However, there was no observed statistically significant difference for the Communicable Diseases Control visit. In addition, no significant differences were detected between the mean responses of male and female students toward all field visits, whether the feedback was provided before or during the COVID-19 pandemic.

**Conclusion:**

Transformation of real public health field visits into virtual format is acceptable and applicable during the COVID-19 era and maybe beyond.

## Introduction

Since December 2019, when the outbreak of severe acute respiratory syndrome coronavirus 2 (SARS-CoV-2) emerged with so far over 405 million reported cases around the world and around 5.7 million deaths (1), online distance learning has mostly replaced face-to-face traditional teaching in closed classrooms (2). While recent reports suggest that the COVID-19 pandemic is expected to reach a plateau with the adaptation of mass vaccinations in most countries and eventually will start to fall (3), virtual learning is likely to continue to rise and may never decline again (4). Educational institutions have discovered that in comparison to classroom traditional face-to-face lectures, teaching *via* the internet in many circumstances appears to be more acceptable to the young digital native generation of students. Virtual learning is reported to increase retention of information and is perceived to be more fun by students resulting in long-lasting changes in the educational environment that are here to stay, not only during the COVID-19 era but most likely even beyond (5, 6). In line with these reforms in the learning experiences of students, similar feedback was reported by medical students with studies on transforming didactic face-to-face lectures into online sessions, workshops, or seminars that appear to be more appealing to medical students (7).

Arabian Gulf University (AGU) is a regional university established in 1980 and is based in the Kingdom of Bahrain. AGU hosts students of both genders from the Gulf Cooperation Council (GCC) countries (Bahrain, Saudi Arabia, Kuwait, Oman, UAE, and Qatar). The College of Medicine and Medical Sciences (CMMS) follows a problem-based, student-centered, and community-oriented curriculum (8). The Community Health Programs are an integral part of the academic activities of the Department of Family and Community Medicine. The programs are based on the department's vision to further integrate public health. Thus, the department has expanded its activities to include more programs by offering a more competitive view of public health as a homeostatic mechanism for the sustainability of the population's health since the academic year 2009–2010. This program enhances the medical students' knowledge of preventive medicine and raises their awareness of the role of public health in maintaining the health of the population (9).

**Figure 1:**
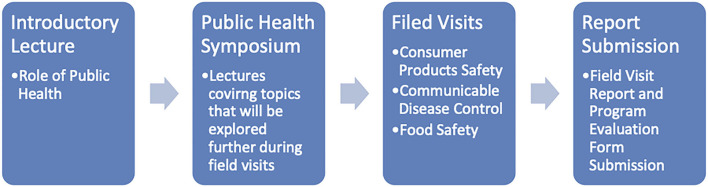
Components of the Public Health Program Activities.

**Figure 2:**
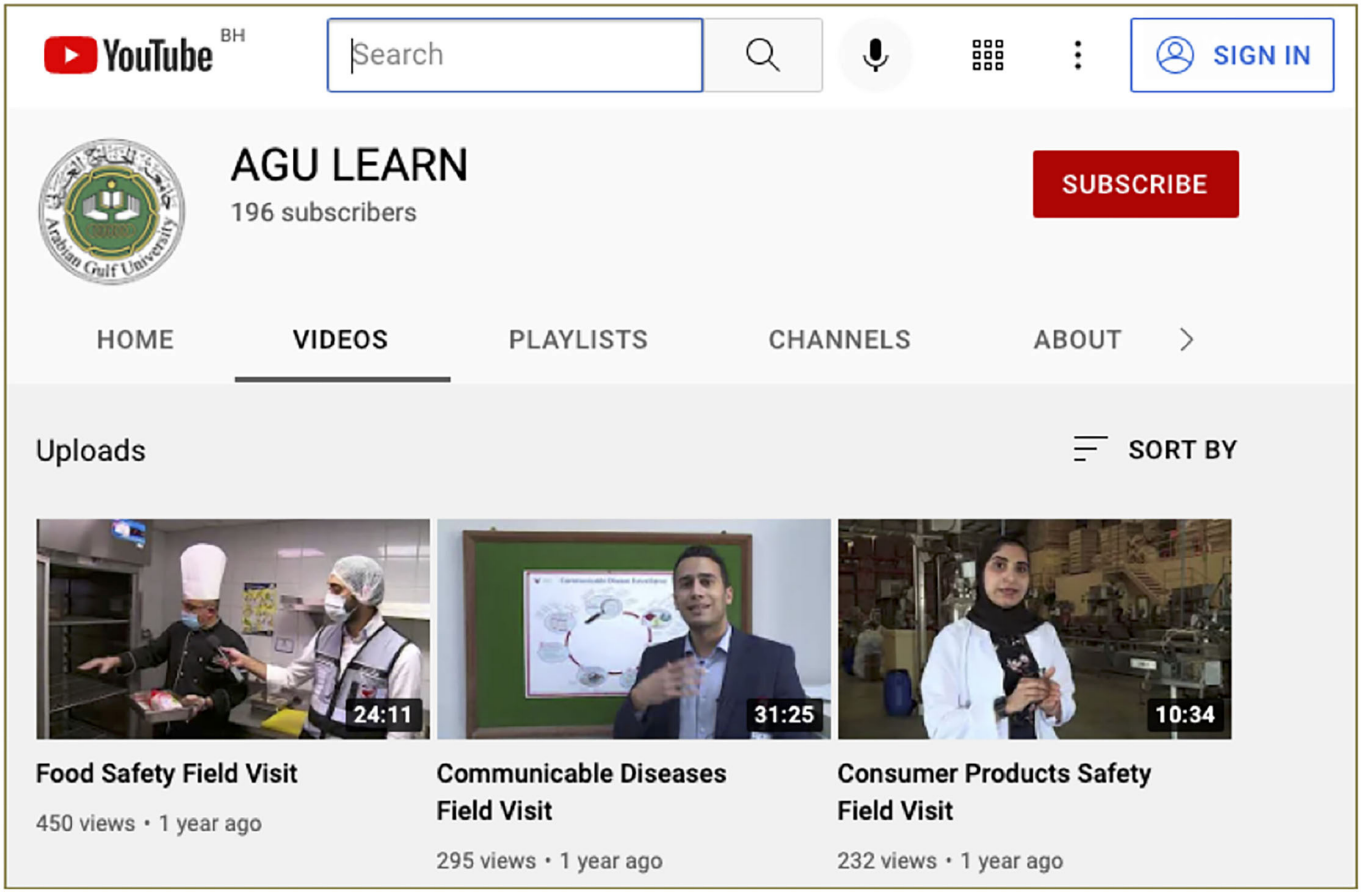
Video-taped Public Health Program Field Visits Videos.

The program begins with an introductory lecture to prepare students for the Public Health Program and familiarizes them with its role in the health of populations. Then students will attend a public health symposium, which is a series of three lectures covering public health topics that will be further explored during field visits. The core component of the program comes next, which is the field visit activity where students visit three different sites within the sections of the Public Health Directorate, which are consumer products safety, communicable diseases control, and food safety. At the end of the program, students are required to submit a field visit report on one of the field visits along with the program's evaluation form (Figure 1). However, during the COVID-19 pandemic and due to social distancing policies, the program was delivered online *via* Moodle learning management system (LMS) while live lectures were delivered *via* Zoom^TM^ (Zoom Video Communications, Inc., San Jose, CA, USA). The field visits' component of the Public Health Program was conducted by faculty and videotaped in a similar format as in the original program (10–12). Subsequently, these recorded visits were delivered to students *via* YouTube^TM^ (YouTube, Inc., San Bruno, CA, USA) in order to fulfill the program requirements (Figure 2). In addition, the program logbooks, field reports, and program evaluation forms were submitted *via* LMS.

Virtual learning technologies are not new and have been adopted by many tertiary education institutes since the turn of the century. However, the COVID-19 crisis has accelerated the transition from pure face-to-face traditional learning into the virtual or hybrid format. Systematic reviews of published educational literature on the effectiveness of virtual medical teaching have generally found wide acceptance from students and faculty to the digital transformation of learning activities as long as there is adequate internet connectivity and accessibility (13–15). In the GCC countries and Bahrain in particular, such services are ranked among the top in the world with 98% of the general population having access to the internet (16). This has obviously facilitated the digital transition of educational activities in the GCC countries during the pandemic. In line with this educational development, most non-clinical programs were shifted into online format at CMMS-AGU. While many studies have explored the effects of the change from face-to-face to virtual format, there is hardly any published research on transforming real field visits into virtual ones, particularly in public health activities. Moreover, studies have mainly addressed the students' perspectives involved with the transition from traditional to distance learning (17). This study is aimed to examine the potential effect of transforming real field visit programs into virtual format during the COVID-19 pandemic. If it is found that the educational concepts are not compromised (based on the views of the students), and given the likely difficulties in attaining access for students to such site visits in the future, then virtual field visits may either replace or at least complement future educational activities.

## Materials and Methods

### Study Population

This descriptive cross-sectional study compared the students' feedback on Public Health Program before and during the COVID-19 pandemic. Feedback evaluation forms were submitted by medical students upon completing the Public Health Program in the academic years 2019–2020 (before the COVID-19 pandemic) and 2020–2021 (during the COVID-19 pandemic). The responses to these forms were analyzed in order to study the difference, if any, in students' perceptions toward this innovative format of the program.

### Study Instrument

An online self-administered questionnaire (Supplementary Material) was developed to assess the students' feedback upon completing the Public Health Program. While the completion of the feedback evaluation form was not mandatory, students were strongly encouraged to submit their responses upon the successful completion of the program. The questionnaire consisted of two main sections: the first one was intended to collect general information, whereas the second part sought students' feedback about Public Health Program before the COVID-19 pandemic and during the pandemic by using ten (10) items. Those items were categorized into three fields: consumer products safety (items 1–4), communicable diseases control (items 5–7), and food safety (items 8–10). The response options of the questionnaire items represented 5 Likert-type scales (1 = very poor, 2 = poor, 3 = average, 4 = good, and 5 = very good). To report the results of this study, we combined “very good” and “good” into one category as satisfied, and “very poor” and “poor” were considered as unsatisfied.

### Sample Size

All medical students who have submitted online feedback evaluation forms upon completing the Public Health Program in the academic years 2019–2020 (180 students; before the COVID-19/ pandemic) and 2020–2021 (167 students; during the COVID-19 pandemic) were included in the study, a total sample size of 347 responses.

### Data Collection

Data were collected based on forms received for the academic years 2019–2020 and 2020–2021.

### Statistical Analysis

Statistical analysis was conducted using the Statistical Package for Social Sciences (SPSS), version 28. The internal consistency and reliability questionnaire was measured by Cronbach's alpha. Categorical variables were presented as frequencies and percentages, whereas continuous variables were presented as mean and standard deviation (SD). Independent samples *t*-test was used to compare students' feedback on Public Health Program before and during the COVID-19 pandemic. Effect size was measured using Cohens d. The conventional effect sizes proposed by Cohen are 0.20 (small effect), 0.50 (moderate effect), and 0.8 (large effect) (18). To further analyze the proportion of students' responses, Likert responses “very good,” “good,” “average,” “poor,” and “very poor” were grouped together into one cohort, this was created into three categorical variables, which were compared using a chi-square test. Cramer's V coefficient was used to measure and interpret the association effect size (0–0. <0.10: negligible association; 0.10– <0.20: weak association; 0.20– <0.40: moderate association; 0.40– <0.60: relatively strong association; 0.60– <0.80: strong association, and ≥0.80: very strong association) (19). A *p*-value of <0.05 was considered as statistically significant.

### Ethical Considerations

This study was approved by the Research and Ethics Committees of the CMMS at AGU (approval number: E43-PI-1-22). The names of students chosen to answer the questionnaire were kept anonymous. All data were kept confidential.

## Results

A total of 347 students participated in the present study. In total, 180 students participated before the COVID-19 pandemic (real field visits, the response rate was 93.3%), and 167 students participated during the COVID-19 pandemic (virtual field visits, the response rate was 88.4%). Most of the participants were women (70.6%), and it reflects the female majority of the undergraduate medical students in AGU. The results related to the internal consistency reliability showed that the Cronbach's alpha coefficient for all items of the questionnaire was 0.95, and for each field Cronbach's alpha coefficients (consumer products safety, communicable diseases control, and food safety) were 0.931, 0.903, and 0.904, respectively, which was considered satisfactory.

### Students' Evaluation Toward Public Health Program According to the Visiting Period

The Pearson chi-square test indicated that there was a significant association between whether the visit was before or during the COVID-19 pandemic period and all the items that fall under the first field, which are consumer products safety regulations and registration (Table 1): [χ^2^ = 18.067, *p* < 0.001], safety and quality of products [χ^2^ = 15.871, *p* < 0.001], control and supervise product consignments imported through ports [χ^2^ = 18.187, *p* < 0.001], and inspection programs on-premises [χ^2^ = 19.781, *p* < 0.001]. The magnitude of these relationships is a moderate association, as evidenced by Cramer's V coefficient.

**Table 1:** Association between public health field visit program and visiting period (before and during the COVID-19 pandemic).

**Field visits/statement**	**Real field visits** **(before COVID-19 pandemic;** ***n*** **=** **180)**		**Virtual field visits** **(during COVID-19 pandemic;** ***n*** **=** **167)**		***p*-value**	**Effect size (Cramer's V)**
	***N***	**%**	***n***	**%**		
**CONSUMER PRODUCTS SAFETY**						
**Consumer products safety regulations & registration program**						
Unsatisfied	22	12.2	13	7.8	<0.001	0.228^*^
Neutral	52	28.9	21	12.6		
Satisfied	106	58.9	133	79.6		
**Examine the safety and quality of products, monitor compliance to standards**						
Unsatisfied	24	13.3	15	9.0	<0.001	0.214^*^
Neutral	47	26.1	19	11.4		
Satisfied	109	60.6	133	79.6		
**Control & supervise product consignments imported through ports**						
Unsatisfied	29	16.1	10	6.0	<0.001	0.229^*^
Neutral	40	22.2	20	12.0		
Satisfied	111	61.7	137	82.0		
**Inspection programs on premises related to consumer products**						
Unsatisfied	24	13.3	11	6.6	<0.001	0.239^*^
Neutral	46	25.6	18	10.8		
Satisfied	110	61.1	138	82.6		
**COMMUNICABLE DISEASES CONTROL**						
**Notification of diseases in Bahrain**						
Unsatisfied	17	9.4	16	9.6	0.070	0.124
Neutral	34	18.9	17	10.2		
Satisfied	129	71.7	134	80.2		
**WHO surveillance guidelines**						
Unsatisfied	22	12.2	16	9.6	0.025	0.146^**^
Neutral	34	18.9	16	9.6		
Satisfied	124	68.9	135	80.8		
**Control of communicable diseases**						
Unsatisfied	17	9.4	11	6.6	0.099	0.115
Neutral	34	18.8	20	12.0		
Satisfied	129	71.7	136	81.4		
**FOOD SAFETY**						
**Food safety Rules and regulations**						
Unsatisfied	20	11.1	13	7.8	<0.001	0.209^*^
Neutral	37	20.6	12	7.2		
Satisfied	123	68.3	142	85.0		
**Inspection of food premises**						
Unsatisfied	22	12.2	10	6.0	<0.001	0.263^*^
Neutral	42	23.3	12	7.2		
Satisfied	116	64.4	145	86.8		
**Prevention and control of food borne diseases**						
Unsatisfied	20	11.1	8	4.8	<0.001	0.217^*^
Neutral	46	25.6	21	12.6		
Satisfied	114	63.3	138	82.6		

* *Moderate association*.

** *Weak association*.

In addition, the results related to the communicable disease control field visit indicated that there was a significant association between the visit period and the satisfaction of medical students related to WHO surveillance guidelines [χ^2^ = 7.418, *p* = 0.025]. The effect size according to Cramer's V coefficient showed that the magnitude of these relationships is a weak association.

Regarding the food safety field visit, the results showed there was a significant association between the visit period and the satisfaction of medical students related to the following: food safety rules and regulations [χ^2^ = 15.136, *p* < 0.001], inspection of food premises [χ^2^ = 23.935, *p* < 0.001], and prevention and control of foodborne diseases [χ^2^ = 16.293, *p* < 0.001]. The magnitude of this relationship is a moderate association for all of these items according to the effect size of Cramer's V coefficient.

Overall, our results showed that in all fields (consumer products safety, communicable diseases control, and food safety), the proportion of students who were satisfied with the Public Health Program during the COVID-19 pandemic period was higher as compared to that before the pandemic (Table 1).

### Comparison of Students' Feedback Toward Public Health Program Before and During the COVID-19 Pandemic

The feedback of medical students regarding their experiences with the Public Health Program showed that the mean response of students during the COVID-19 pandemic was more than the mean response of students prior to the pandemic. Table 2 summarizes the statistically significant differences that were found. The results of the independent samples *t*-test indicated that there were statistically significant differences between the mean response of students before the COVID-19 pandemic and during the COVID-19 pandemic toward each of the field visits to consumer product safety and food safety (*t* = 4.032, *p* < 0.001, Cohens *d* = 0.43) and (*t* = 4.266, *p* < 0.001, Cohens *d* = 0.46), respectively, with medium effect size, which illustrate that students are significantly more satisfied toward each of the consumer product safety and food safety field visits during the COVID-19 pandemic as compared to the students' responses before the pandemic. The results of the field visit of communicable disease control in contrast showed that there were no statistically significant differences between the mean response of students before the COVID-19 pandemic and during the pandemic (*t* = 1.446, *p* = 0.075).

**Table 2:** Comparison of public health field visit program before and during the COVID-19 pandemic.

**Field visits**	**Real field visits** **(before COVID-19 pandemic;** ***n*** **=** **180)**		**Virtual field visits** **(during COVID-19 pandemic;** ***n*** **=** **167)**		***t*-value**	***P*-value**	**Cohen's *d***
	**Mean**	**SD**	**Mean**	**SD**			
Consumer products safety	3.76	1.10	4.22	1.00	4.032	<0.001	0.433
Communicable diseases control	4.05	1.04	4.21	1.01	1.446	0.075	0.155
Food safety	3.88	1.08	4.35	0.95	4.266	<0.001	0.458
Total	3.88	0.97	4.26	0.94	3.631	<0.001	0.390

### Comparison of Students' Feedback on all Items of the Public Health Program

The results related to students' responses toward all the items of the Public Health Program feedback form revealed that the mean score of the response of the students during the COVID-19 pandemic was more than that for students before the COVID-19 pandemic.

Table 3 shows a comparison of students' feedback on all the items of the Public Health Program before and during the COVID-19 pandemic. No statistically significant differences were found between the mean responses of male and female students toward all field visits, whether before or during the COVID-19 pandemic (*p* > 0.05).

**Table 3:** Comparison of students' satisfaction before and during the COVID-19 pandemic according to gender.

**Field visits**	**Real field visits** **(before COVID-19 pandemic)**		***p*-value**	**Virtual field visits** **(during COVID-19 pandemic)**		***p*-value**
	**Male (*n* = 48)**	**Female (*n* = 132)**		**Male (*n* = 54)**	**Female (*n* = 113)**	
	**Mean ±SD**	**Mean ±SD**		**Mean ±SD**	**Mean ±SD**	
Consumer products safety	4.02 ± 1.00	3.67 ± 1.13	0.062	4.05 ± 0.92	4.30 ± 1.03	0.135
Communicable diseases control	4.19 ± 0.92	3.99 ± 1.08	0.264	4.11 ± 0.92	4.26 ± 1.05	0.385
Food safety	4.13 ± 0.90	3.80 ± 1.13	0.071	4.30 ± 0.88	4.38 ± 0.99	0.607
Total	4.10 ± 0.82	3.81 ± 1.00	0.068	4.14 ± 0.83	4.31 ± 0.98	0.282

## Discussion

The COVID-19 pandemic has enhanced the adoption of online distance learning after the suspension of traditional teaching due to closures and lockdowns. Several studies have indicated that theoretical parts of the medical curriculum can be taught *via* virtual strategies while the practical and clinical components would be better delivered *via* either face-to-face or preferably a mixed approach (20, 21). Digital transformation of medical academic activities was mandated during the current COVID-19 pandemic and is likely to become the norm medium of the educational environment even in the post-COVID-19 era.

Students who responded to the online questionnaire have reported significantly higher levels of satisfaction with the modified virtual format of the Public Health Program as compared to the real public health field visits. This finding can be generalized to all students or to student populations of similar socio-demographic structure since the response rate of students was high (93.3% before the COVID-19 pandemic and 88.4% during the COVID-19 pandemic) along with high validity and reliability of the research instrument (Cronbach's alpha of the questionnaire was 0.95. The Cronbach's alpha coefficients of each field visit were above 0.90). It could be argued that the nature of the COVID-19 pandemic and the threat it poses to the whole community may have influenced the opinion of medical students to opt for an alternative method of learning that makes them less prone of being infected with the virus. However, such a limitation would unlikely have significantly affected the students' satisfaction rates with the virtual Public Health Program since the students have already accepted the potential health risk as part of their medical clinical training (21).

The findings of this study show that medical students were more satisfied with the virtual format of the public health field visits in comparison to the real visits. These results are in line with a study that was conducted among medical students in 12 different countries and reported 67% satisfaction with the quantity and 62% satisfaction with the quality of the virtual learning course (22). Similar findings were reported among dental students in Malaysia toward distance learning in comparison to traditional classroom teaching (23). This significant difference was mainly observed in the visits to the consumer products safety and to the food safety facilities, while for the communicable diseases control visit, there was no statistical difference between the real visits (before the COVID-19 pandemic) as compared to the virtual visits (during the COVID-19 pandemic). One possible explanation for this finding is that the students' satisfaction with the communicable diseases control visit was already high, even in the pre-COVID-19 era, and there was little room for improvement. It is also worthwhile to note that both male and female medical students appear to significantly favor the virtual format of the visits more than the real version. These significant differences are unlikely to be a reflection of the social or cultural preferences of students since if such differences were genuinely present; they would have favored the female students. This provides further support to the notion that students were actually more satisfied with the modified virtual format of the visits as compared to the original real visits format. The findings of the study are in line with the recent World Bank Group meta-analysis assessing the effect of virtual training on students learning, which reported that virtual training appears to be more effective than the traditional approach, particularly in the health field (24).

Despite the relatively high satisfaction rate among medical students toward virtual public health field visits in this study, medical training needs face-to-face experiences and student-tutor interactions, which will still be limited in virtual learning. It is worthwhile to note that many tertiary education programs, particularly in engineering, have already accepted the new norm in learning as a mixture of hybrid and virtual learning (25, 26). However, within the context of learning public health concepts, we believe that hands-on training along with virtual learning would be more suitable. We propose, in line with the recommendations from other studies (21, 27) to encourage educators to use hybrid strategies in order to improve the experiences of medical students in learning public health concepts.

In conclusion, virtual public health field visit is an applicable and acceptable learning format, which can be considered along with face-to-face methods. This would be applicable not only in the Gulf Arab population but also in communities with similar social and demographic structures, not only during the COVID-19 era but maybe beyond, in situations where students may be denied access to such public health facilities.

## Data Availability Statement

The raw data supporting the conclusions of this article will be made available by the authors, without undue reservation.

## Ethics Statement

The studies involving human participants were reviewed and approved by Research and Ethics Committees of the College of Medicine and medical science at Arabian Gulf University (approval number: E43-PI-1-22). Written informed consent for participation was not required for this study in accordance with the national legislation and the institutional requirements.

## Author Contributions

SA, AAS, KA-R, and AA: conceptualization, writing—review, and editing and methods. SA, KA-R, and AA: writing—original draft. SA and AA: data curation. AA: data analysis. All authors have reviewed and approved the manuscript. All authors contributed to the article and approved the submitted version.

## Conflict of Interest

The authors declare that the research was conducted in the absence of any commercial or financial relationships that could be construed as a potential conflict of interest.

## Publisher's Note

All claims expressed in this article are solely those of the authors and do not necessarily represent those of their affiliated organizations, or those of the publisher, the editors and the reviewers. Any product that may be evaluated in this article, or claim that may be made by its manufacturer, is not guaranteed or endorsed by the publisher.

## References

[1] World Health Organization. WHO Coronavirus (COVID-19) Dashboard. (2022) Available online at: https://covid19.who.int/ (accessed February 14, 2022).

[2] World Bank. Remote Learning During COVID-19: Lessons from Today, Principles for Tomorrow. (2022). Available online at: https://www.worldbank.org/en/topic/edutech/brief/how-countries-are-using-edtech-to-support-remote-learning-during-the-covid-19-pandemic (accessed February 14, 2022).

[3] World Health Organization. Strategy to Achieve Global Covid-19 Vaccination by mid-2022. (2021). Available online at: https://www.who.int/publications/m/item/strategy-to-achieve-global-covid-19-vaccination-by-mid-2022 (accessed February 16, 2022).

[4] Gaur U Majumder MAA Sa B Sarkar S Williams A Singh K. Challenges and opportunities of preclinical medical education: COVID-19 crisis and beyond. SN Compr Clin Med. (2020) 2:1992–7. 10.1007/s42399-020-00528-1 32984766 PMC7508422

[5] Szymkowiak A Melović B Dabić M Jeganathan K Kundi GS. Information technology and Gen Z: the role of teachers, the internet, and technology in the education of young people. Technol Soc. (2021) 65:101565. 10.1016/j.techsoc.2021.101565

[6] Hernandez-de-Menendez M Escobar Díaz CA Morales-Menendez R. Educational experiences with Generation Z. Int J Interact Des Manufact. (2020) 14:847–59. 10.1007/s12008-020-00674-9

[7] Ramachandran K Kumar RD. Perception of medical students about online learning in the COVID-19 era. Biomedicine. (2021) 41:139–45. 10.51248/.v41i1.54935480544

[8] Hamdy H Anderson MB. The Arabian Gulf University College of Medicine and Medical Sciences: a successful model of a multinational medical school. Acad Med. (2006) 81:1085–90. 10.1097/01.ACM.0000246680.82786.76 17122475

[9] Arabian Gulf University. Public Health Program. Manama: Arabian Gulf University (2021). p .1.

[10] Arabian Gulf University. Consumer Products Safety Field Visit. (2021). Available online at: https://www.youtube.com/watch?v=sS5MisRHGic (accessed February 1, 2022).

[11] Arabian Gulf University. Communicable Diseases Field Visit. (2021). Available online at: https://www.youtube.com/watch?v=f5hvWemdkUA (accessed February 1, 2022).

[12] Arabian Gulf University. Food Safety Field Visit. (2021). Available online at: https://www.youtube.com/watch?v=ppAA-AhGAT8 (accessed February 2, 2022).

[13] Mohtar M Md Yunus M. A Systematic Review of Online Learning during COVID 19: Students' Motivation, Task Engagement and Acceptance. Arab World Eng J (AWEJ) 2nd Special Issue on Covid 19 Challeng. (2022) 2:202–15.

[14] Kusmaryono I Jupriyanto J Kusumaningsih W. A systematic literature review on the effectiveness of distance learning: Problems, opportunities, challenges, and predictions. Int J Educ. (2021) 14:62–9. 10.17509/ije.v14i1.29191

[15] Wilcha R. Effectiveness of virtual medical teaching during the COVID-19 crisis: systematic review. JMIR Med Educ. (2020) 6:e20963. 10.2196/20963 33106227 PMC7682786

[16] Gulf Daily News. Bahrain Among Top 15 Most Internet-Connected Countries. (2018). Available online at: https://www.gdnonline.com/Details/423777/Bahrain-among-top-15-most-Internet-connected-countries (accessed April 13, 2022).

[17] Alkhowailed MS Rasheed Z Shariq A Elzainy A El Sadik A Alkhamiss A. Digitalization plan in medical education during COVID-19 lockdown. Inform Med Unlocked. (2020) 20:100432. 10.1016/j.imu.2020.100432 32959020 PMC7494503

[18] Cohen J. Statistical Power Analysis for the Behavioural Sciences. 2nd ed. Hillsdale, NJ: Lawrence Earlbaum Associates (1988).

[19] Rea LM Parker RA. Designing and Conducting Survey Research: A Comprehensive Guide. San Francisco, CA: John Wiley & Sons (2014).

[20] Muflih S Abuhammad S Al-Azzam S Alzoubi KH Muflih M Karasneh R. Online learning for undergraduate health professional education during COVID-19: Jordanian medical students' attitudes and perceptions. Heliyon. (2021) 7:e08031. 10.1016/j.heliyon.2021.e08031 34568607 PMC8456362

[21] Atwa H Shehata MH Al-Ansari A Kumar A Jaradat A Ahmed J. Online, face-to-face, or blended learning? Faculty and medical students' perception during the COVID-19 pandemic: a mixed-method study. Front Med. (2021) 9. 10.21203/rs.3.rs-951471/v1 PMC8850343 35186989

[22] Stoehr F Müller L Brady A Trilla A Mähringer-Kunz A Hahn F. How COVID-19 kick-started online learning in medical education—The DigiMed study. PLoS ONE. (2021) 16:e0257394. 10.1371/journal.pone.0257394 34547031 PMC8454930

[23] Amir LR Tanti I Maharani DA Wimardhani YS Julia V Sulijaya B. Student perspective of classroom and distance learning during COVID-19 pandemic in the undergraduate dental study program Universitas Indonesia. BMC Med Educ. (2020) 20:1–8. 10.1186/s12909-020-02312-0 33121488 PMC7594975

[24] Angel-Urdinola DF Castillo-Castro C Hoyos A. Meta-Analysis Assessing the Effects of Virtual Reality Training on Student Learning and skills development. World Bank (2021). 10.1596/1813-9450-9587

[25] Khan ZH Abid MI. Distance learning in engineering education: challenges and opportunities during COVID-19 pandemic crisis in Pakistan. Int J Elect Eng Educ. (2021). 10.1177/0020720920988493

[26] Asgari S Trajkovic J Rahmani M Zhang W Lo RC Sciortino A. An observational study of engineering online education during the COVID-19 pandemic. PLoS ONE. (2021) 16:e0250041. 10.1371/journal.pone.0250041 33857219 PMC8049279

[27] Jeganathan S Fleming PS. Blended learning as an adjunct to tutor-led seminars in undergraduate orthodontics: a randomised controlled trial. Br Dent J. (2020) 228:371–5. 10.1038/s41415-020-1332-1 32170259

