# Supplementary material for: Innovations in Medical Education During the COVID-19 Era and Beyond: Medical Students' Perspectives on the Transformation of Real Public Health Visits Into Virtual Format
Source: Front Public Health. 2022 Jun 13;10:883003. doi: 10.3389/fpubh.2022.883003 (PMC9234203; doi:10.3389/fpubh.2022.883003)
Supplement: Supplementary file 1 [file Data_Sheet_1.docx]

Supplementary Material: Questionnaire

**Public Health Program**

Program’s Evaluation Form

**Section 1.**

Student Name: ___________________________ Student ID: ____________

Date: _______________

**Section 2.**

Please tell us your opinion about this program and the extent to which the visits attended were helpful to achieve the learning objectives:

| **Field visit / Item description** | | **Scale** *(Select one)* | | | | |
| --- | --- | --- | --- | --- | --- | --- |
|  |  | 1 | 2 | 3 | 4 | 5 |
| **Consumer Products Safety** | |  |  |  |  |  |
| 1 | Consumer products safety regulations and registration program |  |  |  |  |  |
| 2 | Examine the safety and quality of products, monitor compliance to standards. |  |  |  |  |  |
| 3 | Control and supervise product consignments imported through ports. |  |  |  |  |  |
| 4 | Field inspection programs on premises related to consumer products |  |  |  |  |  |
| **Communicable Diseases Control** | |  |  |  |  |  |
| 5 | Notification of diseases in Bahrain |  |  |  |  |  |
| 6 | WHO surveillance guidelines |  |  |  |  |  |
| 7 | Control of communicable diseases |  |  |  |  |  |
| **Food Safety** | |  |  |  |  |  |
| 8 | Food safety rules and regulations |  |  |  |  |  |
| 9 | Inspection of food premises. |  |  |  |  |  |
| 10 | Prevention and control of food borne diseases |  |  |  |  |  |

***1= Very Poor 2=Poor 3=Average 4=Good 5=Very Good***

Student Signature:
